# The Effects of Frequency and Duration of Handling on the Development of Feline Upper Respiratory Infections in a Shelter Setting

**DOI:** 10.3390/ani10101828

**Published:** 2020-10-08

**Authors:** Charlotte C. Burns, Laurel E. Redding, Brittany Watson

**Affiliations:** 1 Department of Emergency Medicine, MedVet Chicago, 3305 N California Ave, Chicago, IL 60618, USA; charlotte.burns@medvet.com; 2 Department of Epidemiology, University of Pennsylvania School of Veterinary Medicine, 3800 Spruce Street, Philadelphia, PA 19104, USA; lredding@vet.upenn.edu; 3 Shelter Medicine Program, University of Pennsylvania School of Veterinary Medicine, 3800 Spruce Street, Philadelphia, PA 19104, USA

**Keywords:** upper respiratory disease, stress, handling, immune system, cat, shelter

## Abstract

**Simple Summary:**

Stress in cats residing in a shelter environment is a significant contributor to the development of upper respiratory disease (URD). Previous studies have shown that positive interactions can reduce stress and enhance the immune system. However, little is known on how the frequency and duration of daily handling affects a cat’s likelihood of developing URD. The goal of this study was to record the daily frequency and duration of handling of clinically healthy cats that were relinquished at a closed admission shelter and determine if these parameters were associated with a statistically significant increased risk of developing URD. While no parameters were statistically significantly associated with an increased hazard of developing URD, we found that cats that did not develop URD were handled more frequently than cats that did develop URD. Our results suggest that increased frequency and duration of handling does not appear to increase the risk of URD signs and may reduce the incidence of URD development. These results are important for the shelter community in developing handling and hygiene protocols for shelter cats.

**Abstract:**

Reducing stress is important to maintaining the health of shelter cats and decreasing the risk of upper respiratory disease (URD). The aim of this study was to determine if the frequency and/or duration of daily routine handling of shelter cats affects the likelihood of URD development. At a closed admission shelter, each cat free of URD on intake was given a cage card for recording handling data. These data included: date and times when the cat was handled, duration of handling, if and when the cat developed signs of URD, and the handler identity. Cox regression was used to determine the relationship between these factors and URD development. We found cats that did not develop URD were handled significantly more than cats that did (1.1 times per day vs. 0.7 times per day, *p* < 0.001). Increased frequency of handling had a borderline significant effect on the hazard of developing URD (HR 0.37; CI: 0.13–1.1; *p* = 0.066). No other parameters were significantly associated with the development of URD; however, small sample size may be responsible for this finding. A larger study is needed to elucidate the relationship between handling and URD development.

## 1. Introduction

Cats experience stress under various situations. Examples being exposed to novel situations [1], when confined [2], and when receiving medical treatment [3]. Cats in an animal shelter are particularly predisposed to stress [3]: they are surrounded by other animals, handled by unfamiliar people, and kept in confined quarters [4,5,6]. Increased group density of cats in animal shelters is correlated with increased stress [4]. In addition, these types of environments, often due to limited spacing, prevent cats from completing species-typical behaviors such as climbing, predation, exploration, and investigation, leading to increased stress [2]. It has been argued that the medical care provided in shelter settings (spays/neuters, vaccinations, deworming) may inadvertently result in further stress [3]. While one study has shown that cats can acclimate to this new environment in 2–5 weeks [4], another study found that some cats may never adjust to this new form of confinement and may remain stressed for months [6,7]. The diverse origins of cats that enter animal shelters (e.g., owner surrendered vs. feral vs. stray) may also impact their behavior towards human interaction [8].

Unfortunately, when stressful events persist, the immune system can become compromised. Various studies have shown that stimulation of targeted areas in the feline brain that are associated with emotions such as fear, anxiety, and restlessness can alter cell-mediated immunity [9,10,11,12]. These stressful events can also cause immunosuppression through inhibition of mucosal antibodies [9]. Secretory immunoglobulin A is the predominant mucosal antibody involved in mounting an immune response against respiratory pathogens and can be measured in saliva and feces [13,14,15]. Inhibition of this antibody has been correlated to chronic stress in dogs [16], which share a strong mucosal immunity resemblance to cats [13]. Reactivation of underlying diseases, such as feline herpesvirus, has also been correlated with stress [17]. Latently infected carriers of feline herpesvirus have the potential to shed and infect surrounding susceptible cats. Infection can even spread, particularly in catteries and shelters, by indirect transmission via contamination of cages, feeding, cleaning products, and staff [17]. With all of these potential risk factors for spread of disease and immune suppression, there is increased likelihood of developing upper respiratory disease (URD) in shelter cats.

Various studies have been performed to determine if handling of cats in the shelter increases their risk of becoming stressed, with mixed results [18,19]. Some argue even gentle handling can induce aggression [18] either due to genetic predisposition to defensive behaviors towards humans [20,21] or over stimulation of certain epidermal units. The epidermal units (merkel cells, ruffian endings, vibrissae) of the cat discharge rapidly when a cat is under stress, making them sensitive to the touch and thus perpetuate further stress and aggression [18,22]. In contrast, other studies have shown that petting can have numerous positive benefits such as reduction of stress associated with chronic pain [23] and lowering of arterial blood pressure [24]. As it pertains to cats housed in an animal shelter, gentle handling of anxious cats has been shown to decrease stress, increase S-IgA production, and reduce the incidence of upper respiratory disease (URD) [19,25]. As mentioned previously, IgA is the most abundant antibody for mucosal immunity and is vital for the prevention of URD pathogens [13,26].

To our knowledge, no studies have evaluated the relationship between the frequency and duration of routine handling in the shelter and its impact on the development of clinical URD signs. In light of the mixed results reported between stress and handling, this is an important relationship that requires further investigation. Because of the variety of cats entering shelters, an investigation of the impact of generic handling is essential. Understanding the effects of daily routine handling on the incidence of URD development can influence the handling and hygiene protocols of a shelter and ultimately affect the welfare and adoption likelihood of shelter cats. In this study, we hypothesized that increased frequency and/or duration of handling can decrease the hazard of developing URD. 

## 2. Materials and Methods

### 2.1. The Shelter 

The study was conducted at a closed admission animal shelter in Pennsylvania from 3 November 2016 to 29 November 2016. The shelter houses up to 70 cats in three main areas: the intake room (38 spaces), the adoption room (40 spaces), and isolation room (8 spaces). Shelter biosecurity, housing details, and vaccination/deworming protocols can be found in Appendix A. Cats housed in different rooms were exposed to different individuals. Approximately 4–6 employees and 3–4 volunteers work in each cat holding area each day. The public only had access to cats that were in the adoption room. Approximately 40–50 and 80–120 potential adopters visited daily on the weekdays and weekends, respectively.

### 2.2. Animals

All cats were examined on admission into the intake room by veterinary technicians. The physical examination of all cats included: examination of appropriate mentation, hydration status, and palpation of all limbs, abdomen, and dorsum to note any overt wounds or masses. Skin disease, such as flea infestations and ringworm infections, were also noted and treated as needed. Examination of the oral cavity to look for evidence of dental disease or ulceration was also performed. Evidence of URD that was noted by staff such as: conjunctivitis, nasal or ocular discharge, sneezing, congestion, oral ulceration, fever, lethargy, or inappetence was evaluated. Animals determined to have URD by the staff veterinarian’s requirements listed above were started on an oral antibiotic, Doxycycline^a^ by the medical staff. Every animal free of URD signs on admission to the shelter was given a cage card for monitoring and was included in the study. Animals started on only ophthalmic antibiotics, cats that went into foster care, and cats adopted and then returned to the shelter during the pilot study period were excluded from the study.

### 2.3. Monitoring 

**Figure A1:**
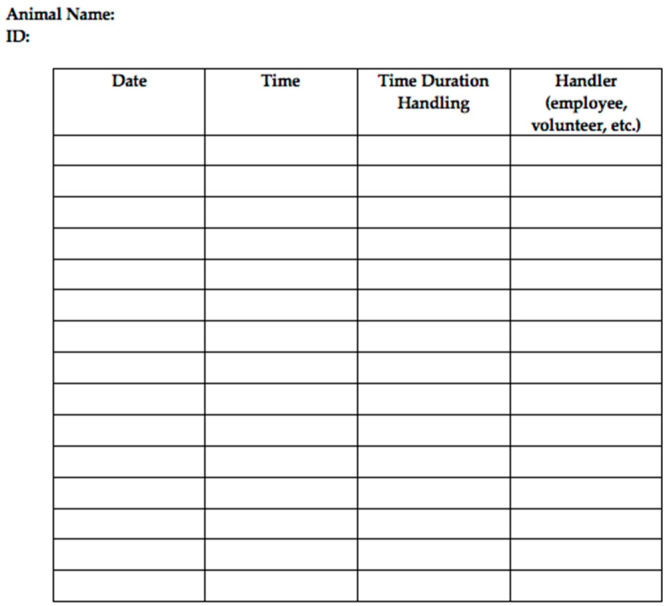
Abbreviated example of a cage card that would be placed on a cage for monitoring the type of handler along with frequency and duration of handling.

On the cage card (Figure A1), the handler marked the date and time when the cat was handled, the duration of handling, and their identity (employee or volunteer). “Handling” was defined as any physical interaction with the cat. This included interacting with the cats during feedings, cleaning, and routine socializations. Cats being anesthetized for surgery were counted to be handled only when they were conscious and able to fully respond to handling. Cats were examined for signs of URD daily by staff. Any URD signs and oral antibiotic therapy prescribed by the shelter veterinarian were noted on the cage card. The following data were extracted from these cage cards: age, sex, whether the animal was altered, length of time in the study (days), total number of times handled during the study, total amount of time handled during the study (minutes), the type of handler (i.e., employee only, volunteer only, or both), and health status (i.e., free of URD or with URD). The origin of the cat (i.e., owner surrender, stray, transfer from another shelter, or picked up by animal control) was also recorded for each cat in the study. 

### 2.4. Statistical Analysis 

Fisher’s exact and χ^2^ tests were used to compare categorical variables (sex, altered status, handler, origin) among the two groups of cats (free of URD vs. with URD). Then, *t*-tests were used on normally distributed continuous variables and Wilcoxon rank-sum tests were used on non-normally distributed continuous variables (age, length of stay in the shelter, total number of times handled, total amount of time handled). A Poisson regression model was used to determine the average number of times each patient was handled per day. Because cats were in the shelter for variable periods of time and with an increased length of stay would be at greater risk of developing URD (due to increased stress) [27,28] along with being observed to have the endpoint of interest (i.e., cats that left the shelter but developed URD outside of the shelter would not have been categorized as having the endpoint of interest), we performed survival analysis to examine the association between handling and the outcome of development of URD. Cats that developed URD while at the shelter were considered to have experienced the endpoint of interest. All other cats were censored when they left the shelter or at the end of the study period, whichever came first. First, univariable analyses were performed to identify parameters that were significantly associated with the outcome, including sex, age, altered status, type of handler, length of stay in the shelter, average number of times handled per day, and origin of the cat. Variables that were significantly associated with the outcome were then added to a multivariable Cox model in stepwise fashion and retained in the final model if they remained significantly associated with the outcome. A *p*-value equal to or less than 0.05 was considered significant. All analyses were performed with STATA^b^ statistical analysis software (StataCorp, College Station, TX, USA).

## 3. Results

A total of 79 cats were included in the study, 28 (35%) of which developed URD and were placed on an oral antibiotic treatment during the study period. Cats that were not included in the data analysis included one cat that was placed into isolation (for which handling data was not collected), and one cat with inadequate recording of handling. 

The median (range) age of cats analyzed in this study was 7 (1-107) months. There was no significant difference in age or sex between the cats that developed URD and those that did not (*p* = 0.488 and 0.097, respectively) (Table 1). Ninety-two percent (71/77) of cats included in the data analysis were identified as domestic short hair (DSH) and therefore the effect of breed was not analyzed. Of the 77 cats included in the data analysis, 56/77 (72.7%) were transferred from nearby shelters, 13/77 (16.8%) were owner surrenders, 6/77 (7.8%) were brought in by humane law enforcement, and 2/77 (2.6%) were relinquished as strays. In comparison to the cats transferred from surrounding shelters, there was no significant difference in the development of URD signs regardless of whether the animal was a stray or brought in by humane law enforcement (*p* = 0.53, *p* = 0.34, respectively). Cats that were owner surrendered had a borderline significant increased hazard of developing URD signs relative to cats that were transferred (HR 2.44; CI: 0.95–6.25; *p* = 0.062). 

The overall average (+/− SD) length of stay (LOS) for all cats was 10.3 days (+/− 6 days), 13 days (+/− 6.9 days) for the cats that developed URD, and 9 days (+/− 5 days) for the cats that did not develop URD, and this difference was statistically significant (*p* = 0.004). The mean (+/− SD) length of time between intake and development of URD was 7.2 (+/− 4.8) days. While on average cats were handled for similar amounts of time at each handling (7.7 min/handling non URD cats vs. 7.4 mins/handling URD cats, *p* = 0.76), cats that did not develop URD were handled almost twice as many times per day as cats that did develop URD (1.1 times per day vs. 0.7 times per day, *p* < 0.001, Median 1.3, IQR 1.1-1.5) (Table 2).

No factors were found to be significantly associated with the development of URD on Cox regression (Table 1). The average number of times handled per day bordered on being significantly associated with development of URD with each additional handling event associated with a 63% decrease in the hazard of developing URD (HR 0.37; CI: 0.13–1.1; *p* = 0.066). The proportional hazards assumption was not violated for this model. Cox–Snell residuals showed good model fit.

## 4. Discussion

The major findings in our study were that URD-free cats were handled significantly more frequently in comparison to cats with clinical signs of URD despite comparable lengths of stay, and this increased frequency resulted in a borderline significant decrease in the hazard of developing URD. In contrast to other studies, we considered all handling equivalent, regardless of when the handling took place, the type of handling, and who was doing the handling. Stratification of handling may have identified differential effects of different types of handling on the development of URD, but we did not collect these data. While we did not look specifically at just positive human interactions (e.g., gentling), the results from our study are a practical extrapolation from the conclusions of Gourkow et al. 2014 [19]. This study found that positive human interaction, also called gentling, can reduce the incidence of URD in shelter cats. While this was a thorough and well-designed study, the ability to apply the methods and findings of this study is limited for most shelters. Training staff to recognize and perform positive handling requires dedicated time and resources. Informing staff that increased handling does not significantly worsen URD development may be a more practical alternative. Overall, we felt that obtaining data from routine handling would be valuable to serve as a baseline to begin to understand how and to what degree standard shelter handling practices affect the feline shelter population.

The relationship between handling and increased immunity is unknown and thus requires further investigation. As mentioned previously, Gourkow et al. 2014 [19] found that cats that experienced gentle handling had increased secretion of IgA thus increasing mucosal immunity. Similar findings have also been noted in other species. In rats, gentle stroking was found to decrease arterial blood pressure [29]. Studies in dogs [30] have shown that gentle petting increases oxytocin release, which has various benefits on overall health [31,32,33,34]. 

It is important to remember the possibility that cats that did not develop URD signs were handled more than cats that did develop URD signs due to other contributing factors. It is possible that the staff or public either consciously or unconsciously avoided cats that appeared clinically sick. Cats themselves might also have been less likely to interact if feeling sick, or sick cats might have been moved to a different location that allowed less contact. Concerns for the cat’s welfare, signaling from the cat it did not want to be disturbed (i.e., actively avoiding handling, sleeping more), or wanting to avoid exposing other cats in the shelter are all possibilities that could have led to cats with URD signs being handled less.

An interesting finding that warrants further exploration was the relationship between the origin of the cat and the development of URD. We found that cats that were owner surrendered had a borderline increased hazard of developing URD signs relative to cats transferred from surrounding shelters. This finding was noted in a previous study [35], which argued that cats that were owner surrendered were less likely to be exposed to URD pathogens and thus be more vulnerable. In contrast, Gourkow et al. 2013 [36] found that cats relinquished to the shelter as strays had an increased cumulative probability of developing URD signs in comparison to cats that were owner surrendered. The relationship between the origin of cats and development of URD requires further investigation. No other patient signalment information such as age, sex, or altered status was found to be significantly associated with the development of URD in this study. However, other studies have noted that some of these factors, such as age and altered status have been correlated with increased development of URDs. Bannasch and Foley 2004 noted that there was increased risk of URD development in cats between the ages of 0–3 months and 7–11 months, but reduced risk for cats greater than 12 months of age [37]. In terms of altered status, Gourkow et al. 2013 noted that the cumulative probability of developing URD was 0.5 times as great for altered cats in comparison to intact cats [36]. However, Edwards et al. 2008 argues that this may be contributed to the fact that more owner surrendered cats are altered [35].

In addition, it was also found in our study that cats that stayed at the shelter longer were more likely to develop signs of URD compared to cats that were adopted sooner. Increased length of time in the shelter has been correlated with increased risk of URD in numerous studies [27,28]. Possible reasons for this finding include reactivation of latent feline herpesvirus infections (FHV-1) due to stress [17], increased risk of being moved to a new cage which some believe complicates or increases the risk of disease [28,37], or just increased risk of exposure to a higher volume of other felines [38,39]. 

A major limitation of this study was that many cats were lost to follow-up prior to the end of the study. The mean time to developing URD was 7.2 days, and 40% of cats were followed for 7 days or less due to being adopted, fostered, or euthanized. We could only assess the effect of handling on the hazard of developing a URD during the time these cats were in the shelter. This loss to follow-up and differences in length of stay likely resulted in a loss of power to detect an effect of handling on the development of URD. This loss to follow up also could have formed a selection bias in that cats without URD may have been handled more and possibly adopted sooner, enriching our sample with either cats with clinical signs of URD or soon-to-be clinically sick cats. Lost to follow up is difficult to control for in an active shelter without holding animals longer than needed. Lastly, it is important to note that this pilot study was performed in a short time period and during the late fall/early winter which may have contributed to our small sample size.

Another limitation of this study is that given its retrospective nature, many cats in this study were missing data recordings, which could have affected our results. For example, if cats that developed URD were less likely to have their handling data recorded, then increased handling would incorrectly appear protective against URD. Finally, all handling was considered to be equivalent—whether the cat was held for a prolonged period of time, played with, fed, etc. Different types of handling could result in varying physiological responses. Presuming a short simple interaction (e.g., opening the cage door to feed the cat) could achieve a similar beneficial effect as a prolonged, tactile interaction (e.g., petting a cat) is likely unrealistic. Behavior in response to handling may have also been valuable information, but it was not collected in this study.

Likely differences in vaccine history could also impact disease development. Cats that were transferred from previous facilities or picked up by humane law enforcement could have had previous vaccinations that could have heightened their immunity to feline URD. In our study the majority of the sample population were cats transferred from other shelter facilities, increasing the likelihood that many of the cats had some form of protective immunity prior to admission to the shelter. PCR testing for feline URDs, as was performed in Wagner et al. 2018 [40], would have been a useful tool, but unfortunately this was not possible at this particular shelter.

There are several future studies that could be performed to further elucidate the findings in this study. An example would be a prospective study that compares standard handling practices to various increased frequencies of handling of shelter cats to determine if there is any significant difference in the development of clinical URD signs. Another idea would be to incorporate animal location within the shelter as location may affect handling practices. One could also consider dividing cats into groups of specific handlers to see if there is any significant difference in URD development if a cat is handled solely based on one individual or group of individuals (i.e., employee vs. volunteer). Lastly, comparing handling practices at different types of shelters (closed admission vs. open admission) may aid in determining if a shelter’s resources and facilities influence the results found in this study. 

## 5. Conclusions

We found that cats that did not develop URD were handled significantly more than cats that did develop URD and this increase in frequency of handling had a borderline significant effect on the reduction of URD development. These results suggest that increased frequency of handling does not increase the risk of URD development and may in fact mitigate the risk of URD in shelter cats. Future studies with a larger sample size and stratification of types of handling are warranted for further clarification of the relationship between feline handling and URD development in the shelter.

## Figures and Tables

**Table 1:** Effect of various factors on the hazard of developing upper respiratory disease in cats staying at a shelter. *p*-value < 0.05 considered significant.

Factor	Hazard Ratio	Confidence Interval	*p*-Value
Female	0.60	0.27–1.34	0.22
Age (months)	0.99	0.98–1.00	0.36
Altered status	0.44	0.13–1.50	0.19
Handler	0.78	0.48–1.07	0.102
Average length of time handled (min)	0.99	0.89–1.1	0.96
Average number of times handled per day	0.37	0.13–1.1	0.066

**Table 2:** Comparison of various average time variables from the Poisson regression model between cats that developed clinical signs of upper respiratory disease (URD) vs. those that did not develop URD signs. *p*-value < 0.05 considered significant. Significance identified with *.

Factor	URD	Non-URD	*p*-Value
Average length of stay (days)(+/−SD)	13 (+/−6.9)	9 (+/−5)	0.004 *
Average length of time handled (min)	7.4	7.7	0.76
Average number of times handled per day	0.7	1.1	<0.001 *

## Appendix A

### Appendix A.1. Shelter Floorplan

Cats at the shelter were housed in either three rooms: intake, isolation, or the general adoptions room. Cats housed in intake are kept in individual stainless-steel cages (shore-lines). There are two banks of cages that are 5 feet across from each other that line opposite walls. In the general adoption room, there is a mix of shore-line single individual cages, group housing, and free roaming cats. Lastly, in the isolation room, there is a single bank of stainless-steel shore-line cages that line a single wall.

### Appendix A.2. Biosecurity

Cages in each area of the shelter were cleaned daily. During cleaning, all eliminations, newspaper, and litterboxes are removed and replaced with clean material, and cage walls and floors are cleaned with Rescue Disinfectant^c^ at a 1:32 dilution. After being disinfected, each cage is furnished with newspaper, a towel for bedding, a litterbox with shredded newspaper, and stainless-steel metal bowels for food and water. Spot cleaning was performed daily. Paper towels are used for the removal of debris. Cages were deep cleaned (i.e., soaked for 10 min) when a cat left or entered a new cage.

The prevention of cross contamination between cats varies based on the room in the shelter. Cats in intake and isolation are handled only by shelter staff with gloves. Staff were required to change gloves between each cat. Cats remain in the intake room for an average of 3–6 days and 10 days in insolation. Staff who are assigned to these rooms are not allowed to transfer between rooms during their shift. Cats in the general adoption are allowed to free roam so sanitation is maintained by requiring all volunteers and potential adopters to sanitize or wash their hands between cats.

### Appendix A.3. Vaccination/Deworming Protocol

#### Vaccine Protocol

On intake cats (>6 months of age) and kittens (4 weeks to 6 months) are examined by the animal care staff and a staff veterinarian is notified if they have medical concerns. Healthy cats weighing at least 16 ounces were vaccinated with an FVRP vaccine and receive a booster FVRCP in 2 weeks if they are still in the shelter. Kittens are vaccinated with FVRCP and are vaccinated every 2 weeks until they are 16 weeks. Rabies vaccination is given to any cat or kitten over 12 weeks of age or weighing at least 48 ounces. The FVRCP vaccine is administered subcutaneously in the left hind leg. The rabies vaccination is administered in the right hind leg. A single Intra-nasal Bordetella vaccination is given to all cats and kittens that are at least 4 weeks of age.

### Appendix A.4. Deworming Protocol:

Kittens are dewormed with pyrantel pamoate^d^ every 2 weeks until they are 16 weeks. Two doses of Ponazuril^e^ are also administered to kittens every 2 weeks. Cats are dewormed with pyrantel pamoate^d^ if they have no prior medical history. Another dose of pyrantel pamoate^d^ is re-administered in 2 weeks.

### Appendix A.5. Feeding Protocol

Cats are fed ¼ cup of dry food twice daily with additional wet food and treats when available. The type of food being fed depends on what is donated to the shelter. Water is available ad libitum.

- Doxycycline, Pfizer, New York, NY, USA.
- STATA 14, StataCorp, College Station, TX, USA.
- Rescue Disinfectant, American Health Packaging, Columbus, OH, USA.
- Pyrantel pamoate suspension, Columbia Laboratories, Lexington, KY, USA.
- Ponazuril, Boehringer Ingelheim, Ingelheim am Rhein, Germany.

## References

[1] Levine S., Moberg G.P. (1985). A definition of stress?. Animal Stress.

[2] Landsberg G. (1996). Feline behavior and welfare. J. Am. Vet. Med. Assoc..

[3] Kry K., Casey R. (2007). The effect of hiding enrichment on stress levels and behaviour of domestic cats (*Felis sylvestris catus*) in a shelter setting and the implications for adoption potential. Anim. Welf..

[4] Kessler M.R., Turner D.C. (1999). Effects of density and cage size on stress in domestic cats (*Felis silvestrus catus*) housed in animal shelters and boarding catteries. Anim. Welf..

[5] Rochlitz I., Podberscek A.L., Broom D.M. (1998). Welfare of cats in quarantine cattery. Vet. Rec..

[6] McCobb E.C., Patronek G.J., Marder A., Dinnage J.D., Stone M.S. (2005). Assessment of stress levels among cats in four animal shelters. J. Am. Vet. Med. Assoc..

[7] McCune S. (1994). Caged cats: Avoiding problems and providing solutions. CABTSG Newsl..

[8] Dybdall K., Strasser R., Katz T. (2007). Behavioral differences between owner surrender and stray domestic cats after entering an animal shelter. Appl. Anim. Behav. Sci..

[9] Gourkow N., LaVoy A., Dean G.A., Phillips C.J.C. (2014). Associations of behaviour with secretory immunoglobulin A and cortisol in domestic cats during their first week in an animal shelter. Appl. Anim. Behav. Sci..

[10] Kojima K., Mohamed S., Fujimaru Y., Mori Y., Kaname H., Sumida Y., Kinukawa N., Tashiro N. (2000). Effects of both the emotional behavior and feeding conditions on the circulating plasma volume and plasma glucose levels in cats. Auton. Neurosci..

[11] Mori Y., Ma J., Tanaka S., Kojima K., Mizobe K., Kubo C., Tashiro N. (2001). Hypothalamically induced emotional behavior and immunological changes in the cat. Psychiatry Clin. Neurosci..

[12] Kaname H., Mori Y., Sumida Y., Kojima K., Kubo C., Tashiro N. (2002). Changes in the leukocyte distribution and surface expression of adhesion molecules induced by hypothalamic stimulation in the cat. Brain Behav. Immun..

[13] Stokes C., Waly N. (2006). Mucosal defence along the gastrointestinal tract of cats and dogs. Vet. Res..

[14] Rodriguez C., Blanch F., Romano V., Saborido N., Rodenas J., Polo J. (2007). Porcine immunoglobulins survival in the intestinal tract of adult dogs and cats fed dry food kibbles containing spray-dried porcine plasma (SDPP) or porcine immunoglobulin concentrate (PIC). Anim. Feed Sci. Technol..

[15] Harley R., Gruffydd-Jones T.J., Day M.J. (1998). Determination of salivary and serum immunoglobulin concentrations in the cat. Vet. Immunol. Immunopathol..

[16] Berteselli G., Servida F., Doll’Ara P., Verga M., Piola E., Puricelli M., Palestrini C., Mills D., Levine E., Ladsberg G., Horwitz A.E., Duxbury A.E. (2005). Evaluation of immunological, stress and behavioural parameters in dogs (*Canis familiaris*) with anxietyrelated disorders. Current Issues and Research in Veterinary Behavioral Medicine.

[17] Gaskell R., Dawson S., Radford A.D., Thiry E. (2007). Review article Feline herpesvirus. Vet. Res..

[18] Rodan I. (2010). Understanding Feline Behavior and Application for Appropriate Handling and Management. Top. Companion Anim. Med..

[19] Gourkow N., Hamon S.C., Phillips C.J.C. (2014). Effect of gentle stroking and vocalization on behaviour, mucosal immunity and upper respiratory disease in anxious shelter cats. Prev. Vet. Med..

[20] McCune S. (1995). The impact of paternity and early socialisation on the development of cats’ behaviour to people and novel objects. Appl. Anim. Behav. Sci..

[21] Reisner I.R., Houpt K.A., Erb H.N., Quimby F.W. (1994). Friendliness to humans and defensive aggression in cats: The influence of handling and paternity. Physiol. Behav..

[22] Overall K.L. (1997). Normal Feline Behavior. Clinical Behavioral Medicine for Small Animals.

[23] Robertson S.A., Lascelles B., Duncan X. (2010). Long-term pain in cats. J. Feline Med. Surg..

[24] Slingerland L.I., Robben J.H., Schaafsma I., Kooistra H.S. (2008). Response of cats to familiar and unfamiliar human contact using continuousdirect arterial blood pressure measurement. Res. Vet. Sci..

[25] Gourkow N., Fraser D. (2006). The effect of housing and handling practices on the welfare, behavior, and selection of domestic cats (*Felis silvestrus catus*) by adopters in an animal shelter. Anim. Welf..

[26] Edinboro C.H., Janowitz L.K., Guptill-Yoran L., Glickman L.T. (1999). A clin-ical trial of intranasal and subcutaneous vaccines to prevent upper respiratory infection in cats at an animal shelter. Feline Pract..

[27] Gourkow N., Lawson J.H., Hamon S.C., Phillips C.J.C. (2013). Descriptive epidemiology of upper respiratory disease and associated risk factors in cats in an animal shelter in coastal western Canada. Can. Vet. J..

[28] Pedersen N., Sato R., Foley J., Poland A. (2004). Common virus infections in cats before and after being placed in shelters with emphasis on feline enteric coronavirus. J. Feline Med. Surg..

[29] Kurosawa M., Lundeberg T., Ågren G., Lund I., Uvnäs-Moberg K. (1995). Massage-like stroking of the abdomen lowers blood pressure in anesthetized rats: Influence of oxytocin. J. Auton. Nerv. Syst..

[30] Odendaal J.S.J., Meintjes R.A. (2003). Neurophysiological correlates of affiliative behaviour between humans and dogs. Vet. J..

[31] Handlin L., Nilsson A., Ejdebäck M., Hydbring-Sandberg E., Uvnäs-Moberg K. (2012). Associations between the psychological characteristics of the human-dog relationship and oxytocin and cortisol levels. Anthrozoos.

[32] Uvnäs-Moberg K., Bruzelius G., Alster P., Bileviciute I., Lundberg T. (1992). Oxytocin increases and a specific oxytocin antagonist decreases pain threshold in male rats. Acta Physiol. Scand..

[33] Uvnäs-Moberg K., Ahlenius S., Hillegaart V., Alster P. (1994). High doses of oxytocin cause sedation and low doses cause an anxiolytic-like effect in male rats. Pharmacol. Biochem. Behav..

[34] Yang L., Carter S., Marucha P.T., Engeland C.G. (2010). Oxytocin speeds wound healing in stressed mice. Brain Behav. Immun..

[35] Edwards D.S., Coyne K., Dawson S., Gaskell R.M., Henley W.E., Rogers K., Wood J.L.N. (2008). Risk factors for time to diagnosis of feline upper respiratory tract disease in UK animal adoption shelters. Prev. Vet. Med..

[36] Bannasch M.J., Foley J.E. (2004). Epidemiologic evaluation of multiple respiratory pathogens in cats in animal shelters. J. Feline Med. Surg..

[37] Gaskell R., Povey R. (1977). Experimental induction of feline viral rhinotracheitis virus re-excretion in FVR-recovered cats. Vet. Rec..

[38] Binns S.H., Dawson S., Speakman A.J., Cuevas L.E., Gaskell C.J., Hart C.A., Morgan K.L., Gaskell R.M. (1999). Prevalence and risk factors for feline Bordetella bronchiseptica infection. Vet. Rec..

[39] Binns S.H., Dawson S., Speakman A.J., Cuevas L.E., Hart C.A., Gaskell C.J., Morgan K.L., Gaskell R.M. (2000). A study of feline upper respiratory tract disease with reference to prevalence and risk factors for infection with feline calicivirus and feline herpesvirus. J. Feline Med. Surg..

[40] Wagner D.C., Kass P.H., Hurley K.F. (2018). Cage size, movement in and out of housing during daily care, and other environmental and population health risk factors for feline upper respiratory disease in nine North American animal shelters. PLoS ONE.

